# Information Persistence Services Designed to Support Home Care

**DOI:** 10.2196/medinform.3699

**Published:** 2015-03-10

**Authors:** Nelson Pacheco Rocha, Alexandra Queirós, Filipe Augusto, Yosvany Llerena Rodríguez, Carlos Cardoso, José Miguel Grade, João Quintas

**Affiliations:** ^1^Instituto de Engenharia Electrónica e Telemática de AveiroHealth Sciences DepartmentUniversity of AveiroAveiroPortugal; ^2^Instituto de Engenharia Electrónica e Telemática de AveiroHealth Sciences SchoolUniversity of AveiroAveiroPortugal; ^3^MaisisMaisisAveiroPortugal; ^4^Instituto de Engenharia Electrónica e Telemática de AveiroUniversity of AveiroAveiroPortugal; ^5^Instituto Pedro NunesCoimbraPortugal

**Keywords:** long-term care, home help services, personal health record, electronic health record, information management, interoperability

## Abstract

**Background:**

Due to the challenges faced by health and social care systems, in particular those related to actual demographic trends, home care emerges as a potentially cost-effective solution to answer the needs of citizens, and to allow the reallocation of resources to alternatives to hospitalization or institutionalization.

**Objective:**

Home care services require cooperation between different actors, including health and social caregivers, care receivers, and their informal caregivers (eg, relatives or friends), across time, space, and organizational boundaries. Therefore, it is foreseeable that eHealth services can contribute to their improvement. The aim of this study is to evaluate information persistence services based on the Reference Information Model (RIM) of the Health Level Seven (HL7) version 3 to support formal caregivers, both health and social care providers, and informal caregivers in the context of home care services.

**Methods:**

A pilot study was set up involving two Portuguese institutions that provide home care services for the elderly. Defining of information requirements was performed according to a comprehensive process. This included a review of the literature, observations of work activities, interviews with caregivers, care receivers and their relatives, analysis of paper documentation related to care receivers’ histories, health conditions and care plans, and brainstorming groups involving specialized professionals. Following this, information objects were implemented and validated.

**Results:**

The methodological approach, as well as the information persistence services, proved to be robust and adequate to specify, implement, and validate different types of information objects related to home care services for the elderly. This study also reinforces the application of the RIM of the HL7 version 3 beyond the strict scope of health care, allowing the persistence of not only health care information, but also information related to social assistance activities.

**Conclusions:**

This study contributes to the ongoing efforts related to the development of eHealth applications to improve the cooperation among formal health care and social caregivers, as well as care receivers and their informal caregivers.

## Introduction

### Background

Health and social care systems need to face challenges due to actual demographic trends, particularly the shift in the burden of illness from acute to chronic conditions. Home care emerges as a potentially cost-effective solution to meet the needs of citizens and to allow the reallocation of resources to alternatives to hospitalization or institutionalization [1]. Home care services can be defined as home-based health care and social care services provided by formal and informal caregivers. This care is not solely focused on clinical purposes, but also on a range of essential activities for the maintenance of the individual's quality of life and that are part of the normal everyday life of every citizen, with the use of technology when appropriate [1-3].

Various interacting organizational structures may coexist within home care services: formal care networks, including both health and social caregivers, and a diversity of informal care providers, such as relatives, friends, voluntary groups, or nongovernmental organizations [1,4]. The introduction of eHealth [5] services can mobilize synergies between the various stakeholders [4].

In order to contribute to solutions to integrate information objects, including the ones commonly associated with the provision of health care, as well as the ones associated with social assistance or that result from the proactive participation of the care receivers or their informal caregivers, the authors developed the We.Can platform [6]. This platform aims to support care in the community and comprises a set of eHealth applications, development tools, and technological services. In particular, these include security mechanisms (eg, authentication, authorization, or confidentiality) and information persistence services (ie, technological services able to retrieve, manage, and deliver information related to care receivers and to guarantee its internal and external semantic interoperability) based on the Reference Information Model (RIM) of the Health Level Seven (HL7) version 3 [7].

This paper deals specifically with the specification, implementation, and validation of information objects using the aforementioned persistence services in a pilot study involving two Portuguese institutions that provide home care services for the elderly. The introductory part of this paper justifies the need for new information objects for this application domain and compares existing solutions to retrieve, manage, and deliver information related to care receivers, for instance, electronic health records (EHRs), personal health records (PHRs), and electronic social records (ESRs). Additionally, this paper describes a pilot study where an implementation of the RIM of the HL7 version 3, together with the dual-model approach of the openEHR [8], was used to support home care services. Results from the pilot study show that the information services that were developed, based on the RIM, can contribute to the persistence of information usually distributed among EHR, PHR, ESR, and special purpose systems, thereby minimizing integration and interoperability issues. We hope this study can contribute to ongoing efforts to develop eHealth applications to support formal and informal caregivers who do not have access to structured information regarding their care receivers.

### Related Work

Since health and social care systems need to adequately meet new demand patterns, eHealth services can contribute to better allocation and management of the available resources, in accordance with the needs of citizens and of the organizations providing care services. However, most existing eHealth services have been developed with the prevalent paradigm of the discrete specialization of clinical activities and, therefore, there is the need to overcome their fragmentation [9]. Concepts such as Medicine 2.0 [10], connected health [11], or holistic health [12,13] promote citizen-oriented and holistic solutions to manage mutual awareness and shared objectives among care receivers and formal and informal caregivers [12]. This requires an effective cooperation not only among health care and social care providers, but also among formal and informal caregivers, which is complex due to the lack of unified models, concepts, and terminologies. Therefore, the challenge exists to develop transversal information objects (ie, information objects to comply with wellness issues and to accommodate information generated by the care receivers or their informal caregivers, including information resulting from innovative monitoring devices [12]) with meanings that must be the same regardless of the organizational, logistical, or cultural differences of the actors involved. Consequently, the information persistence services should be able to contribute to the systematization of models, concepts, and terminologies and to combine new and existing types of information objects to allow coherent information support for each care receiver [12].

Nowadays, the need for retrieving, managing, and delivering large amounts of health care information is being met by EHRs [14]. EHRs, in their simplest form, consist of electronic files containing clinical information about individuals and can help to personalize care, prevent medical errors, promote the consistency of care, refer the correct service providers, control costs, and promote clinical research [15].

However, health conditions are influenced by factors distributed across different levels of impact that interact with each other continuously and in subtle ways [16]. These include behavioral (eg, medication adherence), social (eg, activities and participation), or environmental factors [17]. Nowadays, these factors can be monitored by ambient assisted living (AAL) and mobile health solutions [18-20], which produce a considerable amount of data.

Additionally, PHRs include information related to the individual's lifetime and the record of care maintained by the individuals [8]. This stands in contrast with EHRs, which are operated by organizations and contain information entered by formal caregivers.

The definition and implementation of ESRs have been considered during the last few years [21,22]. ESRs should be composed of various types of information, namely forms (eg, assessment forms used locally or nationally), coded information (mainly for management and statistical reporting purposes), or unstructured information (eg, letters or notes of meetings). Since the service models employed by health and social caregivers are different, there are important differences between EHRs and ESRs [21]. Health care records focus on a single patient, often in considerable detail and depth. On the other hand, social care records place the individuals in their daily living context of family and other informal caregivers, and include their attitudes and the effects on each [21].

Within the eHealth sphere [10,23], the use of technological solutions to provide information services to mediate among different actors that build functional care around the care receiver has been proposed by various authors [12,24-27]. This has been based on different technologies [28-32], including the management of chronic diseases [32-34]. Furthermore, there is a considerable effort to develop suitable services to manage psychosocial information [21,22,35,36], information generated by the care receivers and their informal caregivers, and information resulting from automatic data collection about individuals and their environments, through innovative monitoring devices [19,20,37]. However, the aggregation of data from different sources, and both the coherence and interoperability of the resulting information, require further research [12,38].

## Methods

### Overview

In Portugal, public services represent only a small part of the support that is given to the elderly. Social solidarity institutions (SSIs) (eg, church-sponsored charity institutions called Misericórdias or private social solidarity institutions), as they are in closer proximity to society, take on a very important role in filling the gaps left by public services [39]. SSIs provide a wide range of services, not only with respect to social assistance, but also to health care, and have differentiated employees, including health care personnel and social workers. Part of SSI financial support is regulated by collaboration contracts or agreements with the National Health Care System and, as their importance to long-term care has been increasing, they have become essential to the provision of health and social care.

SSI interventions are grouped by social responses, which are organized according to the needs of the potential care receivers. In Portugal, nearly half of all the social responses of the SSIs are focused on the elderly population and are classified into the following types: (1) social center (to support sociocultural and recreational activities), (2) day care (to help the elderly to stay in their environments), (3) retirement home (to provide social assistance activities, including temporary or permanent accommodation and provision of food, comfort, and hygiene), (4) residence (to provide common apartments to be used by the elderly with partial or total autonomy), (5) foster care (to temporarily or permanently integrate elderly people with technically-qualified families), (6) temporary reception center (to support social emergency situations), (7) night center (to support elderly with autonomy who carry out their activities of daily living at home, but during the night may need some support due to reasons of isolation), and (8) home care services. In evolutionary terms, home care services have been considered as an alternative to more traditional responses, such as retirement homes, and their availability has substantially increased during recent years.

This study deals with the definition of information objects related to home care services for the elderly. A pilot study was set up involving two SSIs that provide home care services, and a comprehensive process was performed to determine the information requirements. This process was based on the Contextual Design methodology [40] and included the following: a review of the literature, observations of work activities, interviews with caregivers, care receivers and their relatives, analysis of paper documentation related to care receivers’ histories, health conditions and care plans, and brainstorming groups involving specialized professionals. Following this, the We.Can platform, which is briefly described in the next section, was used as a tool to implement and validate the information objects that were identified.

### The Technology Used

Since the main goal of the We.Can platform is to provide eHealth applications related to health and social care in the community, it offers flexible structures to ensure the persistence of the information related to care receivers. In order to achieve this, it follows the dual-model approach that has been considerably developed by openEHR promoters [8]. In terms of the information model, the RIM [7], defined by HL7 version 3, was adopted to facilitate interoperability with external information repositories.

The current implementation of the information model follows the Representational State Transfer architectural style to promote greater scalability and is supported by PostgreSQL technology, which was selected based on performance criteria. The current implementation provides comprehensive operations to access the database of the information repository (ie, create, read, and update operations). Note that the delete operation was not considered because the persistence services should not support the deletion of previously existing records, due to the need to guarantee auditing procedures. Therefore, the operations include versioning mechanisms.

From the point of view of information persistence operations, a specific eHealth application should not have to know the structure of the RIM or the structures of the underlying database, but only the high-level structures of the information objects being used. This means that the create, read, and update operations include mechanisms to map each information object instantiation to the structures of the RIM and of the underlying database. These mechanisms ensure consistency with the RIM while avoiding poor optimized records (eg, a large number of empty fields). Additionally, a syntax specifically designed to allow for the formulation of flexible queries was implemented.

**Figure 1:**
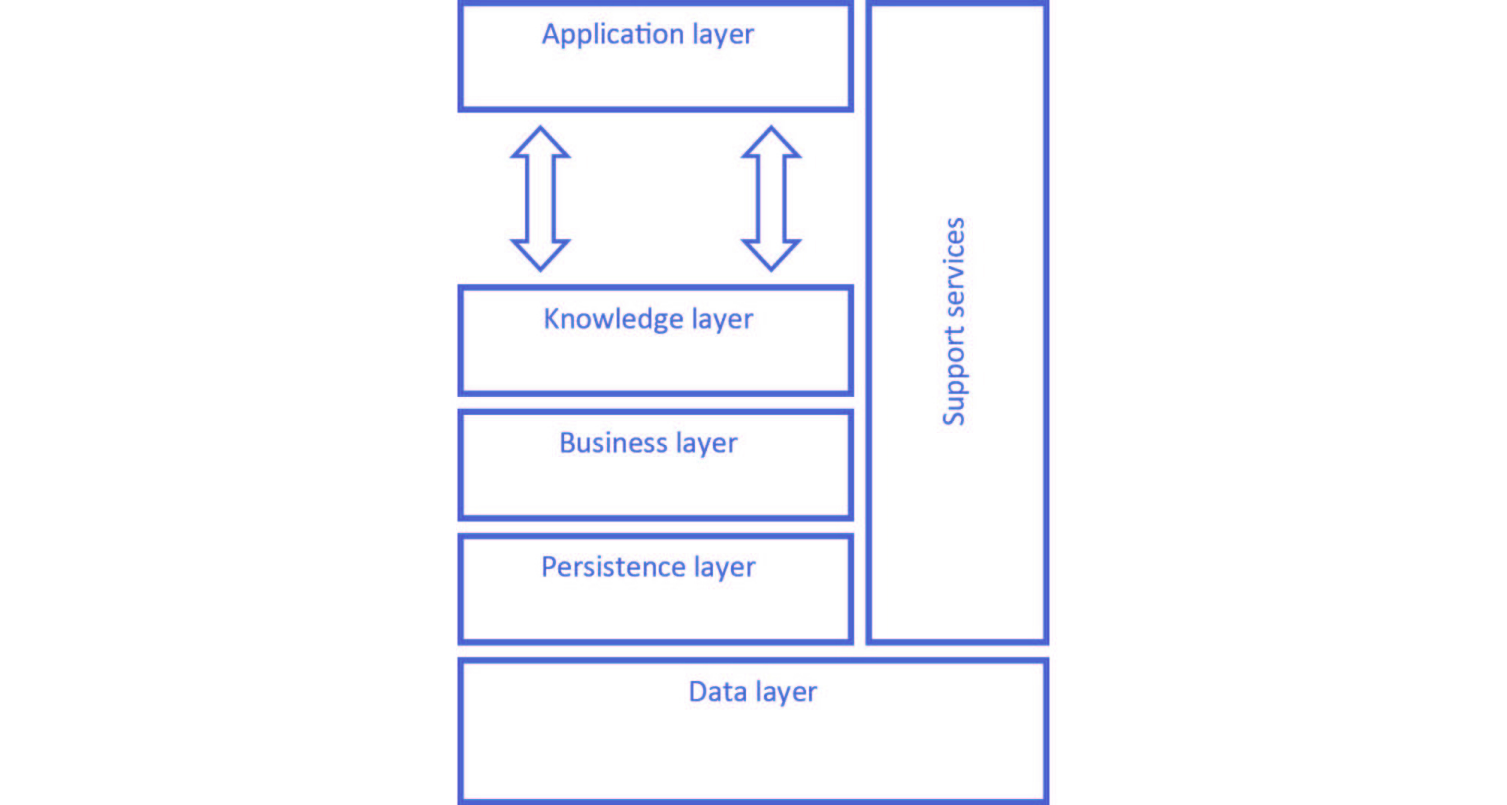
Architecture of the We.Can platform.

A high-level view of the implemented architecture is presented in Figure 1. Briefly, the Persistence layer implements the information model according to the RIM while the Data layer includes the underlying database. The Business layer provides services to ensure the use of the information repository with security and reliability (eg, data transport or security functions, such as authentication, authorization, or confidentiality, among others). This layer also provides the necessary mechanisms for the conversion of messages, to ensure interoperability in terms of the information that is imported from or exported to external information repositories. The Application layer includes specific eHealth applications and the Knowledge layer comprises the functions required to map these applications to the information repository. Finally, development applications are part of the Support services.

Two different development applications have an important role in the context of this study: the Generic Entity configures how the instances of the information objects are accessed and presented, and the Archetypes Manager supports the archetypes implementation (eg, searching, editing, publishing, authentication, versioning, or ownership).

The Generic Entity is intended to help with the development of high-level interaction modules—to be incorporated into the eHealth applications of the Application layer—by using mechanisms to create, access, or modify instances of information objects without the need to know the details of the Persistence layer, namely the structures of the underlying database. Additionally, based on the structures of the information objects, the Generic Entity can implement the visual representations of these objects, as well as the business rules that govern them. The following features provided by the Generic Entity should be highlighted: (1) abstraction of data access and business rules, (2) customization of fields (eg, data type or validation constraints), (3) automatic update whenever there is a definition of new components, (4) automatic generation of custom forms for viewing, entering, or editing data, (5) automatic generation of programming code to be included in the eHealth applications of the Application layer, and (6) versioning management.

For the implementation of the Archetypes Manager, aimed at the optimization of available resources and tools, it was decided to use the openEHR public domain resources. Obviously, this approach has the advantage of substantially reducing development efforts, particularly the ones related to a broad range of constraints that needed to be implemented. Therefore, archetypes and terminologies that were developed by the openEHR community are available, as well as the possibility to edit existing archetypes or create new ones by using openEHR tools, such as LiU Archetype Editor or Ocean Informatics.

**Figure 2:**
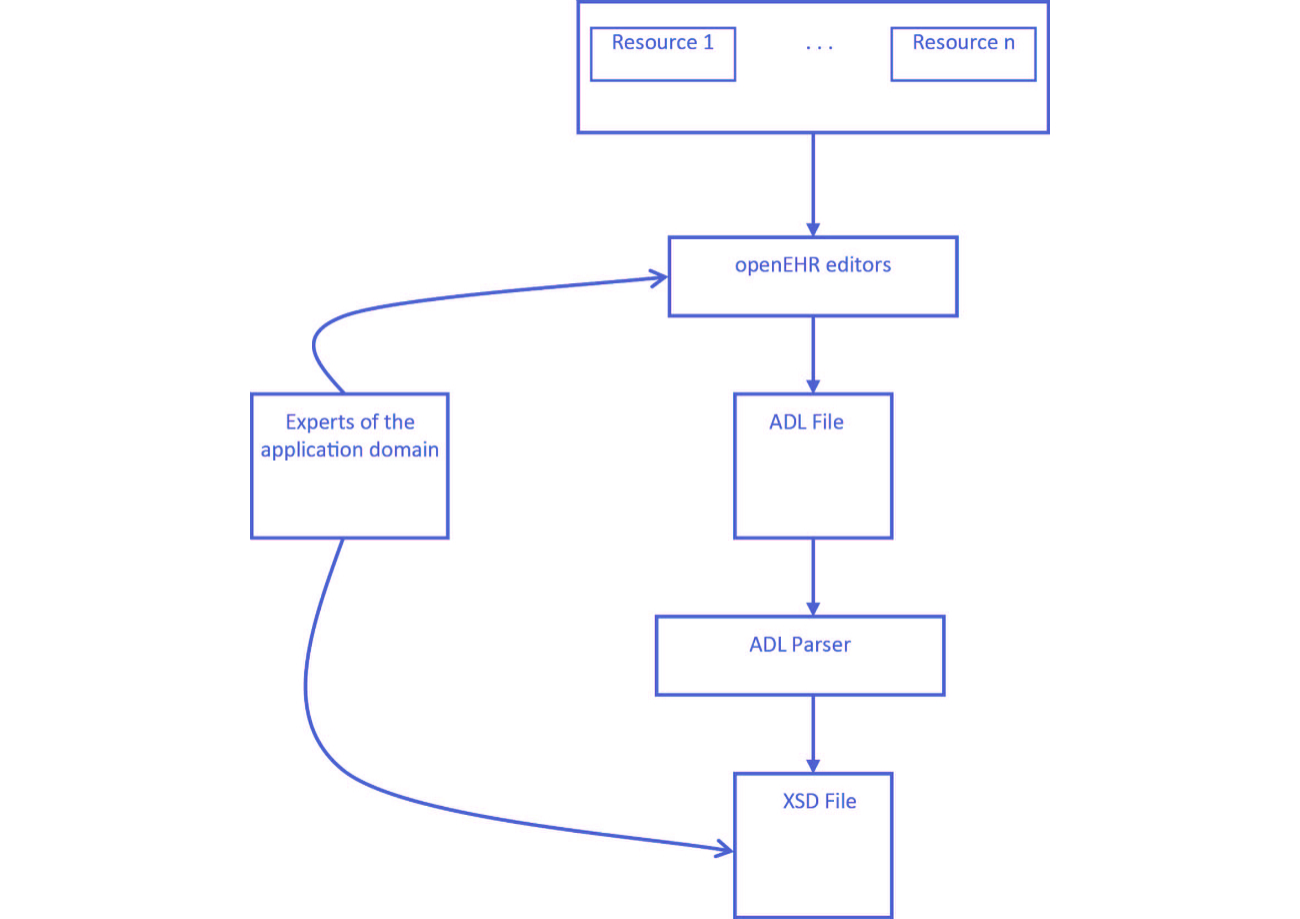
Archetypes Manager.

However, this approach has a drawback due to the inevitable fact that the archetypes resulting from the openEHR tools, described according to Archetype Definition Language (ADL), do not consider the RIM to be the underlying information model. Consequently, bearing in mind the need to map the archetypes’ structures resulting from the openEHR tools, it was necessary to integrate an ADL parser into the Archetypes Manager (Figure 2), this parser then being responsible for all of the necessary mapping procedures.

The formal structures that result from the ADL parser are eXtensible Markup Language (XML) Schema Definition (XSD) files. Within the We.Can platform, these XSD files are used in two different ways: (1) by the Generic Entity to automatically generate programming code related to the high-level interaction modules, including the visual representations and the business rules that govern the respective information objects, and (2) by the applications to invoke the Persistence layer services, and to guarantee the conformity of the instances of the information objects being persisted.

## Results

### Home Care Services

A pilot study was set up involving two Portuguese SSIs that provide home care services for the elderly. These services include the provision of individualized care in the home to individuals when, because of illness, disability, or other impediment, they cannot provide, either temporarily or permanently, the satisfaction of their basic needs or daily living activities [39] (eg, basic health care services, such as rehabilitative, supportive or technical nursing care, and social assistance, such as preparing meals, dressing, hygiene, comfort, home activities, communication, mobility, or transportation).

When potential care receivers, or their relatives, apply for home care services, the applications are evaluated by a committee. For each approved application, an assessment is performed to establish a care plan. This is a key component of home care services and it is based on professional judgments about health conditions and daily living issues.

In organizational terms, there is usually a social worker responsible for home care services who, therefore, takes on the coordination of team leaders and assistants, and interacts with other caregivers (eg, physicians, nurses, psychologists, or therapists).

The team leaders are experienced caregivers and they are responsible for the supervision of the assistants that, in general, do not have differentiated qualifications. The supervision of the assistants during home visits is frequent (ie, at least once a week). The teams can be organized in different ways, depending on the policy of the respective SSI, but they usually work in shifts, with guidelines to ensure that each assistant knows the maximum number of care receivers possible, and to guarantee that house calls are always carried out by two caregivers.

Whenever assistants consider that specific information is relevant, they communicate this to the respective team leader or home care service coordinator, so that appropriate measures can be taken (eg, making an appointment with a general practitioner or asking a specialized technician to give advice on specific assistive technology).

There are formal interactions between the formal caregivers involved, such as briefings about the activities or paper documentation related to care receivers’ histories, health conditions, and care plans. However, the majority of interactions (eg, asking for or sharing information) are informal, face-to-face meetings or phone calls.

### Specification, Implementation, and Validation of Information Objects

**Figure 3:**
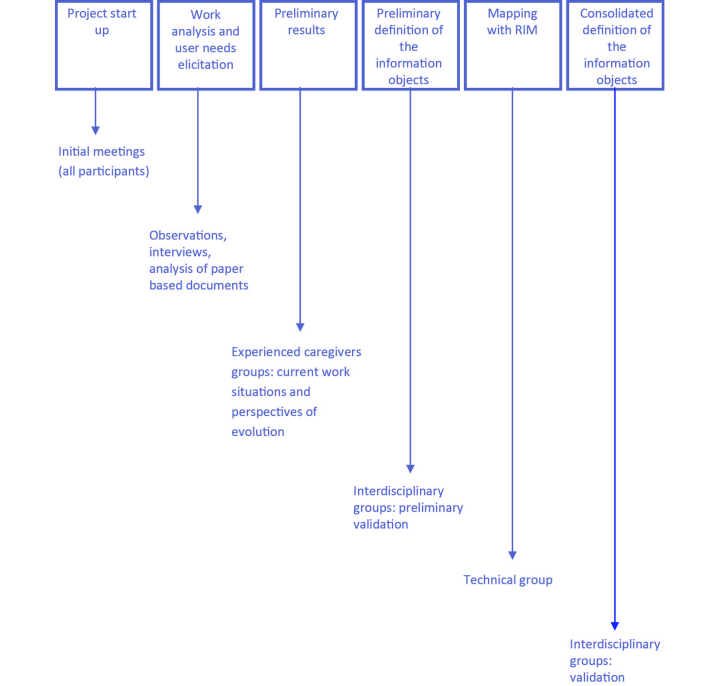
Process to specify, implement, and validate information objects.

After a review of the literature related to home care services, eHealth services and applications, health and social care information, and interoperability standards, a comprehensive process was conducted to specify, implement, and validate the information objects required to support home care services (Figure 3).

Initial meetings were held at the two SSIs to explain the study to all the potential participants. Following these meetings, extensive analyses of work activities and elicitations of user needs were performed based on the Contextual Design methodology [40]. These started with observational studies (ie, observation of the work context and of the activities performed by a number of selected team leaders and assistants during their work shifts). Based on consolidated data of the observational studies, the Unified Modeling Language (UML) was used to model the work activities and the details of the working environment. Additionally, in order to understand the cooperation mechanisms and to include nonobservable aspects of the daily work practices, semistructured interviews focusing on specific objectives, rules, and obligations of the home care services were conducted with various professionals and care receivers and their relatives. Whenever possible, the participants were encouraged to include their personal views and to discuss issues that they thought to be important.

At the same time, an analysis of existing documentation related to care receivers’ histories, health conditions, and care plans was performed. The different documents were studied and categorized according to the type of information they contained (eg, administrative, or health- and social care-related information), to complement the insights from observations and interviews. Different individual files were reviewed until new data could not be extracted from the documents.

The resulting materials were examined repeatedly by members of the research team in order to categorize them, and preliminary empirical results were presented to brainstorming groups. These groups were composed of experienced caregivers who were challenged to develop ideas about information that would be useful to promote person-centered care within home care services (eg, problematic issues, work activities, cooperative activities, information needs, and tools). Additionally, the moderator was instructed to direct discussions, not only toward current work processes, but also to visions of future work practices (eg, AAL or mobile health solutions).

The feedback from the groups composed of experienced caregivers supported the development of a set of archetypes by a team of experts in medical informatics. The resulting archetypes (ie, their formal structure and graphical representation using mind maps) were evaluated by interdisciplinary groups consisting of experienced specialists with different backgrounds (eg, elderly care or medical informatics), aiming as much as possible to encompass a broad perspective of home care services.

To complete the implementation of the information objects, there was the need to map the defined archetypes with the information repository using the ADL parser of the We.Can platform. Following this, the Generic Entity was used, together with the XSD files resulting from the ADL parser, to iteratively refine computerized prototypes. These were validated by the members of the interdisciplinary groups, considering usage scenarios with reasonably detailed descriptions. Finally, experts in medical informatics validated the consistency of the information being persisted.

Most of the qualified caregivers involved in the sequence of procedures being described recognized that it contributed to the following: (1) increasing the mutual understanding of the entire care process and the roles of the various professionals, (2) improving the services being provided and the internal communication, and (3) answering to the political pressure resulting from the modernization of the public administration.

Nevertheless, we should highlight some difficulties, including the following: (1) the natural resistance to the uncertainty and anxiety inherent to transitions, (2) the perception of a high-level of complexity associated with technological solutions, (3) some lack of recognition of the importance of technological solutions and difficulty in evaluating their qualitative benefits, (4) existence of actors that detain key information of critical processes, (5) low ability to contribute to the design of innovative solutions, (6) expertise directed toward the necessity of dealing with daily problems, and not available for planning and realizing advanced organizational and technological innovations, (7) poor formalization of work activities, with most of them in constant mutation, which is subtle at times, (8) lack of standardized procedures, (9) lack of unified models, which results in the use of different concepts and terminologies between different institutions, and even between care providers within the same institution, and (10) existence of rules, jurisdictions, and regulations that are, at times, contradictory, in addition to different institutional and professional cultures.

Last but not least, although there are a significant number of young professionals with a high digital literacy level and who are receptive to the introduction of new services, a low digital literacy level is common to the majority of the older care providers, particularly the assistants. This is also common to most care receivers, which is foreseen to hinder the design, development, and introduction of eHealth services.

The methodological approach—particularly, the brainstorming groups together with the use of computerized prototypes—proved to be robust and reduced the impact of the above-mentioned difficulties in the definition of the different types of information objects related to home care services.

These objects were divided into the following different classes: (1) administrative (eg, account management, claiming, billing, or authorization), (2) demographic (eg, personal and contact information), (3) personal (eg, PHR objects), (4) current health condition (eg, objects for the description of the care receivers’ current health conditions), (5) activity—to group information related to autonomy (eg, the daily activities that the care receivers are able to handle personally and those which they need help with, such as preparing meals, dressing, hygiene, comfort, home activities, communication, mobility, or transportation), habits (eg, eating or sleeping), or social participation, (6) context (eg, issues such as support networks or housing conditions), (7) care delivery—objects to support the ongoing care needs of an individual (eg, information about medication and prescriptions or risk factors), and (8) documentation, including objects for appointments, scheduling, daily notes, care plans (ie, description of the scheduled interventions during the day), performed activities, data from monitoring devices, guidelines, and irregular events (ie, events that, when occurring, imply the need of nonplanned, collaborative interventions).

The implementation of a set of objects with rather different characteristics, as the ones mentioned above, shows that the persistence services of the We.Can platform are able to retrieve, manage, and deliver information required to support home care services. Nevertheless, the integration of data from monitoring devices, such as the ones related to AAL, requires further development. The current implementation does not support the persistence of information related to sensors that provide continuous data streams.

The next section illustrates the implementation of an information object associated with the 9-item Patient Health Questionnaire (PHQ-9) [41], an assessment instrument identified during the definition of the information requirements. Other possibilities could be considered as examples, but since this particular assessment instrument is used internationally, it facilitates the understanding of the procedures related to the implementation of the information objects.

### The 9-Item Patient Health Questionnaire Information Object

The PHQ-9 has been studied as a useful screening tool for depression and is a clinical instrument of public domain. It is suitable for making criteria-based diagnoses of depressive disorders, as well as being reliable and valid in measuring depression severity and in determining the treatment response [41].

In order to determine a score of depression state, the PHQ-9 instrument comprises nine questions that evaluate how often over the last 2 weeks the patient has been bothered by a set of problems. In addition, there is a question to check if there are any problems that impact the way the person works, takes care of things at home, or gets along with other people. Therefore, the PHQ-9 information object is composed of 11 groups (ie, one group for each question and another one for the score).

**Figure 4:**
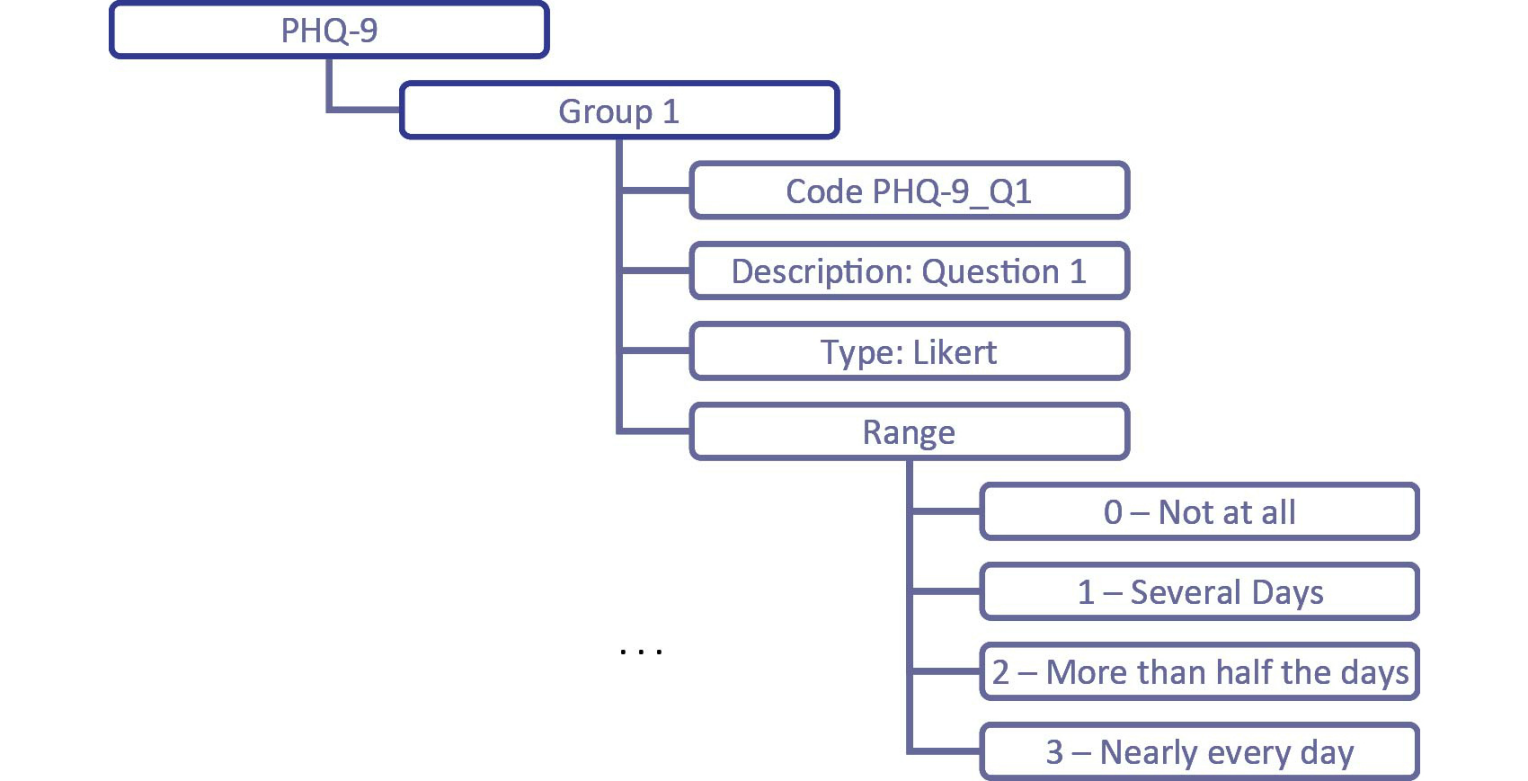
PHQ-9 mind map representation.

Figure 4 represents a mind map related to the specification of the first question that evaluates if the patient has little interest or pleasure in doing things. Since possible answers are *not at all*, *several days*, *more than half the days*, or *nearly every day*, their possible values can be defined using a 4-point Likert scale, with a range from 0 to 3.

**Figure 5:**
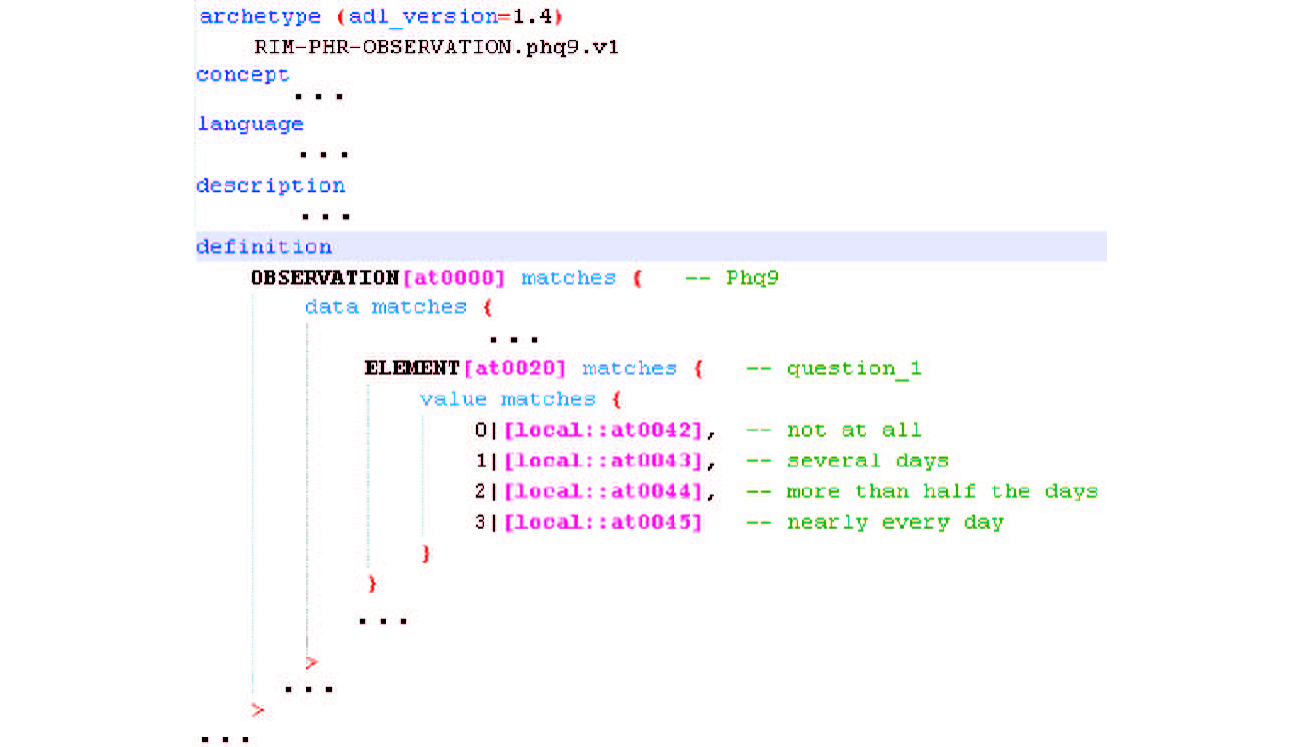
ADL file of the PHQ-9 archetype.

By using an editor provided by the openEHR framework (eg, the LiU Archetype Editor), the information expressed on the mind map can support the specification of the respective archetype. Given the extension of the ADL file resulting from this operation, just a small part (ie, the part related to the first question) is presented in Figure 5.

**Figure 6:**
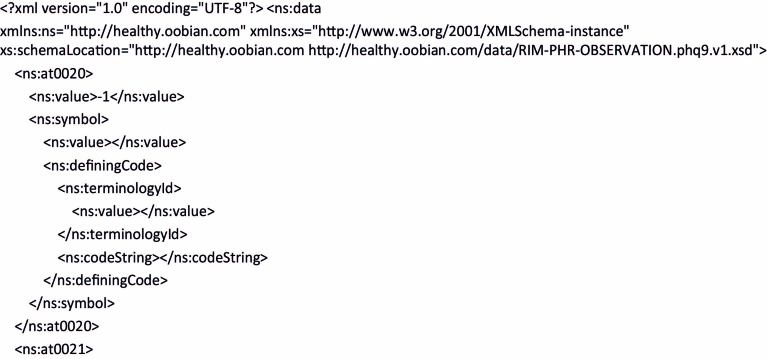
Part of the XML code of the PHQ-9 object.

**Figure 7:**
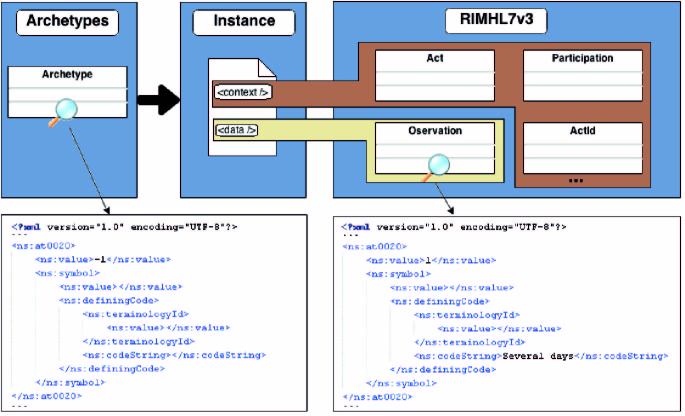
Mapping of a value of the PHQ-9 archetype with an observation value of the RIM structure.

As previously mentioned, an ADL parser was developed. This can be used to create the XSD file required by the applications to guarantee, when invoking the persistence services, conformity with the RIM of all the instances of the PHQ-9 information object. Figure 6 presents part of the XML code resulting from this operation. This figure only focuses on the default value (ie, -1) of the answer to the first question of the PHQ-9. Furthermore, Figure 7 shows a schematic representation of mapping a value of the PHQ-9 archetype with an observation value of the RIM structure.

**Figure 8:**
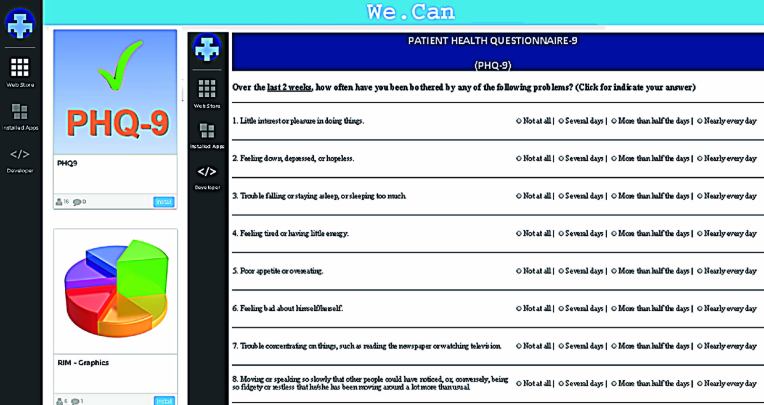
Screen capture of an application prototype to manage the PHQ-9 object.

Finally, the Generic Entity of the We.Can platform and the XSD file were used to generate a high-level interaction module (Figure 8), which was used as a prototype to validate the PHQ-9 information object.

## Discussion

### Principal Findings

Home care services are inevitably complex considering that the provision of care is carried out in the environment of the care receivers, which cannot be easily changed, and that it requires coordination between different actors across time, space, and organizational boundaries [42]. The analysis of work activities related to the home care services of the two SSIs involved in the pilot study shows that most of the information-sharing processes are informal, the institutions tightly control the information related to their care receivers, and the care providers have limited expectations of the information availability.

One of the major findings was the evidence of significant gaps in information availability. Most of the formal caregivers involved in home care services, as well as care receivers and relatives, lacked an overview of the complete care process. For instance, they considered it difficult to access care plans and, therefore, to analyze the interventions performed or being planned.

Despite the complexity of the home care services of the two SSIs involved in the pilot study, the methodological approach that has been followed, as well as the We.Can platform and respective information persistence services based on RIM, proved to be robust and adequate to specify, implement, and validate different types of information objects related to home care services.

### Comparison to Previous Work

Most of the available eHealth solutions to support home care [19,28,32,33] do not present comprehensive approaches for integrating the information related to the care receivers in EHR systems. Frequently, dedicated database solutions are being used for persistence of this information, without considering the available standards for semantic interoperability. However, standards related to interoperability have been considered by the Old@Home project [26,27]. This project has developed tools to support home care services, specifically virtual health records to provide integrated views of information stored in different health information systems [26], and shared care plans to support nurses and social workers in home care services [27]. These shared care plans conform to the European Standard EN 13940-1 for continuity of care, and the Old@Home promoters expressed an interest in evaluating the HL7 standards as a possible alternative [27].

Therefore, this study, by using persistence services based on the RIM to guarantee internal and external semantic interoperability, can contribute to the adequacy of eHealth applications for home care services. This study also shows the application of standards related to health information interoperability beyond the strict scope of health care, allowing the persistence of not only health care information, but also information related to social assistance activities. Particularly in terms of social assistance activities, it would seem that ESRs can be foreseen as future natural extensions of EHRs and PHRs [43], which may contribute to continuous quality improvement [44].

### Limitations

The results of this study, including the methodological approach, should be applied to additional domains. In fact, since the typologies of the care services can be quite different from institution to institution, the results of this study can be enhanced by the experience and knowledge gained from the participation of caregivers and care receivers from institutions offering other types of care services. In particular, integrated care services [9,45] should be considered.

There is a limitation related to the fact that the evaluation of eHealth applications provided by the We.Can platform is still at an early stage. The results presented in this paper are fundamental components for the next phase of the pilot study. This will consist of the evaluation of eHealth applications and a set of information objects to support home care services. Concerning the information objects, further research is required to integrate the broad range of monitoring devices that are available.

### Future Work

The analysis of work activities and the elicitation of user needs—part of the processes related to the specification, implementation, and validation of the information objects—were also used to specify the eHealth applications of the We.Can platform for the pilot study. This specification was based on a detailed description of the different processes of the home care services, such as data collection, initial assessment, care plan definition, preparation of interventions, provision of care, or overall assessment and quality assurance. Given the heterogeneity of the SSIs, these processes are similar up to a certain level, but when details are incorporated they are inevitably different, partly due to organizational, logistical, and cultural differences. The existence of coherent models, such as the one supporting the information persistence services presented in this paper, is important to reduce the impact of these differences and to enable a common view of the processes, procedures, and concepts.

During the period of writing this paper (July 2014), the authors completed the implementation of the eHealth applications. After several working sessions to contextualize how to use these eHealth applications, formal caregivers will be asked to use them, as much as possible, to support their daily work practices. Safeguarding ethical and regulatory issues, information will be taken from volunteer care receivers, including information resulting from monitoring devices, such as the ones able to monitor physiological parameters.

Observations and questionnaires will be used to evaluate the usability and perceived usefulness of the developed eHealth applications, their adherence to the needs and duties related to home care, and their capability of being used in everyday activities to manage the information related to care receivers. The evaluation is planned to take place over several months in order to assess the impact of the eHealth applications and the underlying We.Can platform on the efficiency and effectiveness of home care services.

Following this, the objective of the last phase of the pilot study will be to allow information access not only to formal caregivers, but also to care receivers and their informal caregivers. In terms of research, this will be an opportunity to further develop individualized health and social care services delivery. This includes mechanisms to integrate information provided by formal and informal caregivers, reasonable accommodation of individual choice, efficient teamwork involving formal and informal caregivers, and mechanisms to surpass the difficulties resulting from low levels of digital literacy, which is a major problem considering the target users. In parallel, the technical capabilities of the We.Can platform will be evaluated in terms of conformity, performance, and scalability.

### Conclusions

This study contributes, with integrated solutions, to the persistence of care receivers’ information required to support home care services. This is relevant because the research efforts related to the use of technological services to support individuals in their natural environment should not just consider new ways of collecting information. It should also consider the development of new models and tools that improve information access and interoperability in order to facilitate cooperation among formal health and social caregivers, care receivers, and their informal caregivers, both in technological and organizational terms.

## Abbreviations

AAL: ambient assisted living
ADL: Archetype Definition Language
EHR: electronic health record
ESR: electronic social record
HL7: Health Level Seven
PHQ-9: 9-item Patient Health Questionnaire
PHR: personal health record
QREN: Quadro de Referência Estratégico Nacional
RIM: Reference Information Model
SSI: social solidarity institution
UML: Unified Modeling Language
XML: eXtensible Markup Language
XSD: XML Schema Definition

## References

[1] Genet N, Boerma WG, Kringos DS, Bouman A, Francke AL, Fagerström C, Melchiorre MG, Greco C, Devillé W (2011). Home care in Europe: a systematic literature review. BMC Health Serv Res.

[2] Rosanna T, Tsouros AD (2008). Home Care in Europe: The Solid Facts.

[3] Hofmarcher MM, Oxley H, Rusticelli E (2007). Improved health system performance through better care coordination. OECD Health Working Papers.

[4] Camarinha-Matos L, Ferrada F, Oliveira AI, Rosas J, Monteiro J (2013). Integrated care services in ambient assisted living.

[5] Eysenbach G (2001). What is e-health?. J Med Internet Res.

[6] Rodriguez Y, Cardoso C, Grade M, Augusto F, Queirós A, Quintas J, Rocha NP, Rocha A, Correia AM, Tan F, Stroetmann K (2014). Information persistence architecture for informal and formal care providers. New Perspectives in Information Systems and Technologies, Volume 2.

[7] (2006). Health Informatics -- HL7 version 3 -- Reference Information Model -- Release 1.

[8] Garde S, Chen R, Leslie H, Beale T, McNicoll I, Heard S (2009). Archetype-based knowledge management for semantic interoperability of electronic health records. Stud Health Technol Inform.

[9] Valentijn PP, Schepman SM, Opheij W, Bruijnzeels MA (2013). Understanding integrated care: a comprehensive conceptual framework based on the integrative functions of primary care. Int J Integr Care.

[10] Eysenbach G (2008). Medicine 2.0: social networking, collaboration, participation, apomediation, and openness. J Med Internet Res.

[11] Kvedar J, Coye MJ, Everett W (2014). Connected health: a review of technologies and strategies to improve patient care with telemedicine and telehealth. Health Aff (Millwood).

[12] Rossi Mori A, Mazzeo M, Mercurio G, Verbicaro R (2013). Holistic health: predicting our data future (from inter-operability among systems to co-operability among people). Int J Med Inform.

[13] Koch S (2013). Achieving holistic health for the individual through person-centered collaborative care supported by informatics. Healthc Inform Res.

[14] Häyrinen K, Saranto K, Nykänen P (2008). Definition, structure, content, use and impacts of electronic health records: a review of the research literature. Int J Med Inform.

[15] Eichelberg M, Aden T, Riesmeier J, Dogac A, Laleci GB (2005). A survey and analysis of Electronic Healthcare Record standards. ACM Comput Surv.

[16] Glass TA, McAtee MJ (2006). Behavioral science at the crossroads in public health: extending horizons, envisioning the future. Soc Sci Med.

[17] Alvarelhão J, Silva A, Martins A, Queirós A, Amaro A, Rocha N, Lains J (2012). Comparing the content of instruments assessing environmental factors using the International Classification of Functioning, Disability and Health. J Rehabil Med.

[18] Queirós A, Silva A, Alvarelhão J, Rocha NP, Teixeira A (2013). Usability, accessibility and ambient-assisted living: a systematic literature review. Univ Access Inf Soc.

[19] Rantz MJ, Skubic M, Alexander G, Popescu M, Aud MA, Wakefield BJ, Koopman RJ, Miller SJ (2010). Developing a comprehensive electronic health record to enhance nursing care coordination, use of technology, and research. J Gerontol Nurs.

[20] Reeder B, Meyer E, Lazar A, Chaudhuri S, Thompson HJ, Demiris G (2013). Framing the evidence for health smart homes and home-based consumer health technologies as a public health intervention for independent aging: a systematic review. Int J Med Inform.

[21] Rigby M (2012). Integrating health and social care informatics to enable holistic health care. Stud Health Technol Inform.

[22] Rigby M, Hill P, Koch S, Keeling D (2011). Social care informatics as an essential part of holistic health care: a call for action. Int J Med Inform.

[23] Atkins D, Cullen T (2013). The future of health information technology: implications for research. Med Care.

[24] Bossen C, Christensen LR, Grönvall E, Vestergaard LS (2013). CareCoor: augmenting the coordination of cooperative home care work. Int J Med Inform.

[25] Gusew N, Gerlach A, Bartkiewicz T, Goldapp M, Haux R, Heller U, Hellrung N, Kierdorf HP, Kleinschmidt T, Markurth U, Marschollek M, Plischke M, Schubert R, Seidel C, Wiegmann H (2010). eHealth vision towards cooperative patient care - domain fields and architectural challenges of regional health care networks. Stud Health Technol Inform.

[26] Hägglund M, Scandurra I, Moström D, Koch S (2007). Bridging the gap: a virtual health record for integrated home care. Int J Integr Care.

[27] Hägglund M, Chen R, Koch S (2011). Modeling shared care plans using CONTsys and openEHR to support shared homecare of the elderly. J Am Med Inform Assoc.

[28] Warren I, Weerasinghe T, Maddison R, Wang Y (2011). OdinTelehealth: A mobile service platform for telehealth. Procedia Computer Science.

[29] Stolee P, Steeves B, Glenny C, Filsinger S (2010). The use of electronic health information systems in home care: facilitators and barriers. Home Healthc Nurse.

[30] Ekonomou E, Fan L, Buchanan W, Thuemmler C (2011). An integrated cloud-based healthcare infrastructure.

[31] Meier CA, Fitzgerald MC, Smith JM (2013). eHealth: extending, enhancing, and evolving health care. Annu Rev Biomed Eng.

[32] Li SH, Wang CY, Lu WH, Lin YY, Yen DC (2012). Design and implementation of a telecare information platform. J Med Syst.

[33] Haritou M, Glickman Y, Androulidakis A, Xefteris S, Anastasiou A, Baboshin A, Cuno S, Koutsouris D (2012). A technology platform for a novel home care delivery service to patients with dementia. J Med Imaging Hlth Inform.

[34] Capurro D, Ganzinger M, Perez-Lu J, Knaup P (2014). Effectiveness of eHealth interventions and information needs in palliative care: a systematic literature review. J Med Internet Res.

[35] Glasgow RE, Kaplan RM, Ockene JK, Fisher EB, Emmons KM (2012). Patient-reported measures of psychosocial issues and health behavior should be added to electronic health records. Health Aff (Millwood).

[36] Spitzer WJ, Davidson KW (2013). Future trends in health and health care: implications for social work practice in an aging society. Soc Work Health Care.

[37] Memon M, Wagner SR, Pedersen CF, Beevi FH, Hansen FO (2014). Ambient assisted living healthcare frameworks, platforms, standards, and quality attributes. Sensors (Basel).

[38] Knaup P, Schöpe L (2014). Using data from ambient assisted living and smart homes in electronic health records. Methods Inf Med.

[39] Santana S, Dias A, Souza E, Rocha N (2007). The Domiciliary Support Service in Portugal and the change of paradigm in care provision. Int J Integr Care.

[40] Holtzblatt K, Beyer H (2014). Contextual Design: evolved. Synthesis Lectures on Human-Centered Informatics.

[41] Löwe B, Kroenke K, Herzog W, Gräfe K (2004). Measuring depression outcome with a brief self-report instrument: sensitivity to change of the Patient Health Questionnaire (PHQ-9). J Affect Disord.

[42] Petrakou A (2007). Exploring cooperation through a binder: a context for IT tools in elderly care at home.

[43] Mori A, Dandi R, Mazzeo M, Verbicaro R, Mercurio G, Mot E (2012). Technological solutions potentially influencing the future of long-term care. Enepri Research Report, no. 114.

[44] Spitzer WJ, Davidson KW (2013). Future trends in health and health care: implications for social work practice in an aging society. Soc Work Health Care.

[45] Kodner DL (2009). All together now: a conceptual exploration of integrated care. Healthc Q.

